# The EU Nitrates Directive: A European Approach to Combat Water Pollution from Agriculture

**DOI:** 10.1100/tsw.2001.377

**Published:** 2001-12-12

**Authors:** Gert J. Monteny

**Affiliations:** Institute of Agriculture and Environmental Engineering (IMAG), Wageningen, The Netherlands

**Keywords:** Nitrates Directive, nitrate concentration, Good Agricultural Practice, Vulnerable Zones, animal manure application, nitrogen application

## Abstract

From 1991 onward, the European Union (EU) member states have had to comply with the Nitrates Directive. The aim of this directive is to sustainably protect ground and surface waters from pollution with nitrogen (nitrate) originating from agriculture. Agriculture is, on an EU level, the largest single source of nitrate (runoff, leaching) pollution, although households and industries also contribute to some extent. An important element in the directive is the reporting every 4 years on the monitoring of ground- and surface-water quality. Furthermore, all 15 member states are compelled to designate so-called Nitrate Vulnerable Zones (NVZs). These are regions where the nitrate concentrations in the groundwater amount to 50 mg/l or more. In addition to Codes of Good Agricultural Practice, valid on a countrywide basis and often consisting of voluntary-based measures, specific Action Programmes with mandatory measures have to be developed for the NVZs. The first reporting period ended in 1995. This paper describes the progress in member states’ compliance with the Nitrates Directive during the second period (1996–1999), with a focus on the agricultural practices and action pro- grammes. An evaluation of the member states’ reports shows that good progress is being made on the farmers’ awareness of the need to comply with EU regulations on the protection of the aquatic environment. Action programmes are valuable tools to enforce measures that lead to a reduction of the water pollution by agricultural activities. Regional projects show that significant improvements can be achieved (e.g., reduced fertiliser inputs) while maintaining crop yields and thus maintaining the economic potential of agriculture.

## INTRODUCTION

Nitrogen input through fertilisers (chemical, animal) is larger than the crop uptake in many regions in the European Union (EU). This surplus of nitrogen is discharged, directly or indirectly, to the environment. Important sources of nitrogen pollution by agricultural activities are runoff and leaching, as well as deposition of ammonia upon its volatilisation (e.g., see Burton)[1]. Nitrogen not only causes environmental problems in receiving water bodies but also can affect human health when polluted water is used as a source for the production of drinking water (a comprehensive overview of these effects is presented by Grossman)[2].

To reduce the nitrogen pressure from agriculture to groundwaters and surface waters, the Nitrates Directive, referred to as "directive", was adopted in 1991 by the European Commission[3]. Within the scope of this directive, countries are compelled to designate Nitrate Vulnerable Zones (NVZs). In these NVZs, measures have to be taken to reduce nitrate leaching, resulting in nitrate concentrations in ground and drinking water below 50 mg/l (equivalent to 10 mg/l of nitrate–N; values confirmed by the Council Directive 98/83/EC, issued in November 1998). To achieve this, member states are compelled to formulate and implement Action Programmes for these NVZs. Moreover, according to Article 10 of the directive, member states are required to submit assessment reports to the European Commission every 4 years.

In these reports, the following issues have to be addressed:

- results of the water-quality monitoring activities (groundwater, surface water, coastal waters)
- (progress in) designation of NVZs
- implementation of a Code of Good Agricultural Practice
- implementation of an Action Programme
- forecast in the development of the water quality

The member states' reports on the second reporting period (1996–1999) form the basis for the evaluation presented in this paper. The EU approach to reduce water pollution with nitrates from agriculture is also presented. Emphasis is put on the developments in agricultural practices and action programmes as essential elements of member states' mitigation strategies for water pollution.

## NITRATE VULNERABLE ZONES

NVZs are the key element in the Nitrates Directive and are defined as "known areas draining into waters which are or could be affected by pollution and which contribute to pollution by intensive use of fertilisers or intensive livestock production". Strictly speaking, these areas are likely to contribute to nitrate pollution of surface waters or groundwaters to a level at or above 50 mg/l. In general, these NVZs are regions with high animal densities (large organic and mineral N inputs) or intensive arable crop production (large inputs through mineral N).

Specific action programmes have to be implemented in these NVZs to reduce N pressures and thus nitrate concentrations in water bodies. Table 1 gives an overview of the areas of the 15 EU member states and the NVZs, both for the situation before 1998 ("original") and for the future, with additional NVZ areas based upon a recent EC assessment.

The data in this table show that the total area of the NVZs in the EU-15 amounted to 37% of the total land area. Denmark, Germany, Luxembourg, Netherlands, Austria, and Finland designated their whole territories as vulnerable zones. Furthermore, the aim of the EC is to additionally designate another 9% of the EU area in the near future. The largest extensions are projected in Belgium (+51%), Italy (+19%), Spain (+14%), Portugal (+13%), and Sweden (+10%). These areas all have intensive cropping and/or livestock production.

**TABLE 1**
**Area Per Member State and Per Nitrate Vulnerable Zone (×1,000 km$^2$ and in %) and the Additionally Proposed Vulnerable Zone Area Based on an Assessment Conducted by the European Commission (EC)**

| | | Area Nitrate Vulnerable Zones | | | |
|---|---|---|---|---|---|
| **Member State** | **Total Area** | **Original** | **%** | **Additional** | **%** |
| Belgium | 31 | 2.7 | *9* | 15.9 | *51* |
| Denmark | 43 | 43 | *100* | | |
| Germany | 356 | 356 | *100* | | |
| Greece | 132 | 6.0 | *5* | 9.0 | *7* |
| Spain | 504 | 32 | *6* | 70.6 | *14* |
| France | 539 | 227 | *42* | 37.1 | *7* |
| Ireland | 69 | 0 | *0* | 6.5 | *9* |
| Italy | 301 | 5.8 | *1.2* | 88.7 | *19* |
| Luxembourg | 3 | 3 | *100* | | |
| Netherlands | 37 | 37 | *100* | | |
| Austria | 84 | 84 | *100* | | |
| Portugal | 91 | 0.9 | *1* | 12.2 | *13* |
| Finland | 334 | 334 | *100* | | |
| Sweden | 448 | 41 | *9* | 43.7 | *10* |
| U.K. | 244 | 7.8 | *3* | 19.2 | *8* |
| TOTAL EU-15 | 3,216 | 1,180 | *37* | 303 | *9* |

## Nitrogen Cycle on the Farm Scale and Environmental Impact of Losses

Nitrate pollution is closely related to the agricultural activities. Nitrogen is an essential nutrient for animal and crop production. Losses of N occur in all parts of the farming system. Fig. 1 is a schematic overview of the nitrogen cycle on the farm level for common dairy farming with grassland exploitation. Common N inputs to the system are concentrates and mineral fertilisers. These inputs depend on the farming system and its management. The purchase of organic manure, livestock (cattle), and roughage highly depend on the farming strategy and may not be relevant for all farms. Finally, N inputs through deposition and biological N fixation are common but are beyond the scope of the farmers' management.

Within the farming system, the N cycle consists of N in organic manure (produced during grazing and land-applied manure collected in the house and storage) and N in roughage (in grazing and harvested as silage).

Common N output is through milk and cattle, and in some situations roughage and manure are sold. Finally, N is lost through leaching, denitrification, volatilisation of ammonia ($NH_3$), accumulation in the soil, denitrification of nitrate, and runoff. These sources of N losses are the most important in the framework of the directive.

Excessive leaching of nitrogen, together with oxidation of ammonia to nitrate in the subsoil, is the main cause of increased nitrate concentrations in groundwater. The migration of nitrate through the subsoil is a relatively slow process, so that it may take years or decades before changes in agricultural practices are reflected in the nitrate concentrations. As a result, the nitrogen concentration in the groundwater can increase even when leaching on the field level is reduced. Denitrification and biological fixation of nitrogen will result in a breakdown of nitrate before it reaches the (deeper) groundwater or surface waters.

Volatilisation of ammonia may occur during storage and spreading of animal manure. After oxidation, ammonia will contribute to an increase in the nitrate concentrations when it is deposited in or near water bodies. Gerlach[4] found a threefold increase from 1950 to 1980 of the airborne nitrogen load to be a reasonable estimate for the German coastal waters (from 0.7 t/km2 to 2 t/km$^2$). Hansen et al.[5] estimated an increase of 40% from the 1950s to the 1980s in atmospheric nitrogen deposition to the whole North Sea.

Finally, runoff can occur, which is mainly related to the application of fertilisers close to watercourses, in combination with the geomorphology of the area (e.g., slope), the presence of crops, and weather conditions (precipitation, frost, melting snow). Runoff directly results in an increase of the nitrate concentration of surface water.

Action programmes mainly have to focus on measures that reduce the N pressure on soils (improved balancing of N inputs and N outputs), thus reducing leaching and runoff, although other measures like reduction of ammonia emissions also can be taken into account.

## Major Nitrogen-Related Developments in Agriculture in the EU-15

After the 2nd World War, the use of mineral nitrogen strongly increased (Fig. 2), mainly to maximise the crop production. At

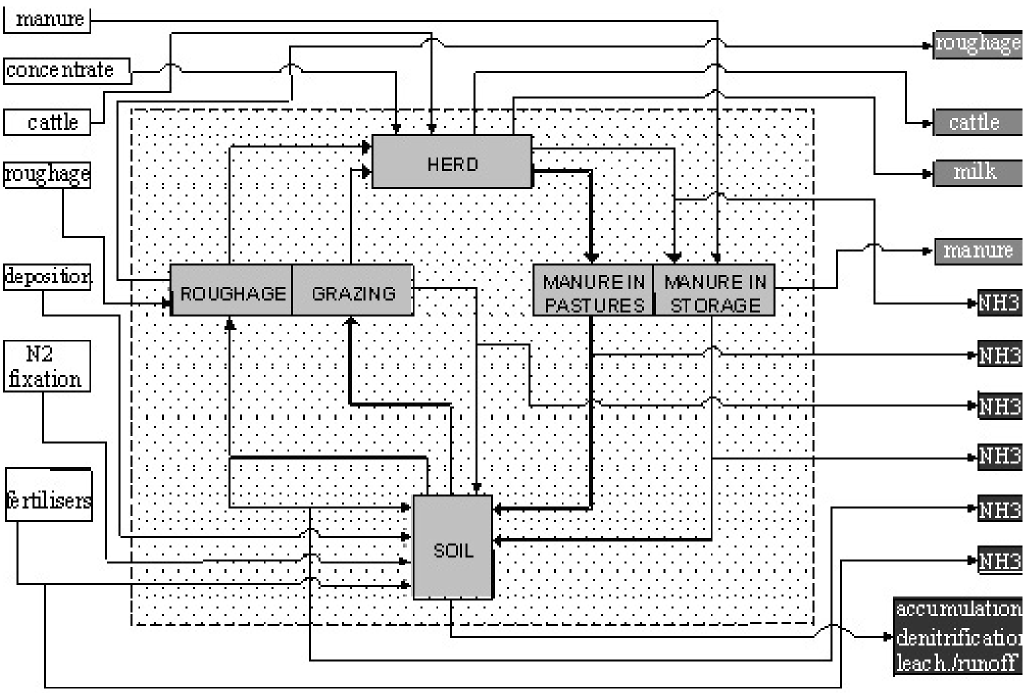

**FIGURE 1**. Nitrogen cycle on farm level with (left hand side) inputs to the (central part) farming system and (right hand side) nitrogen output and losses (Source: Plant Research International).

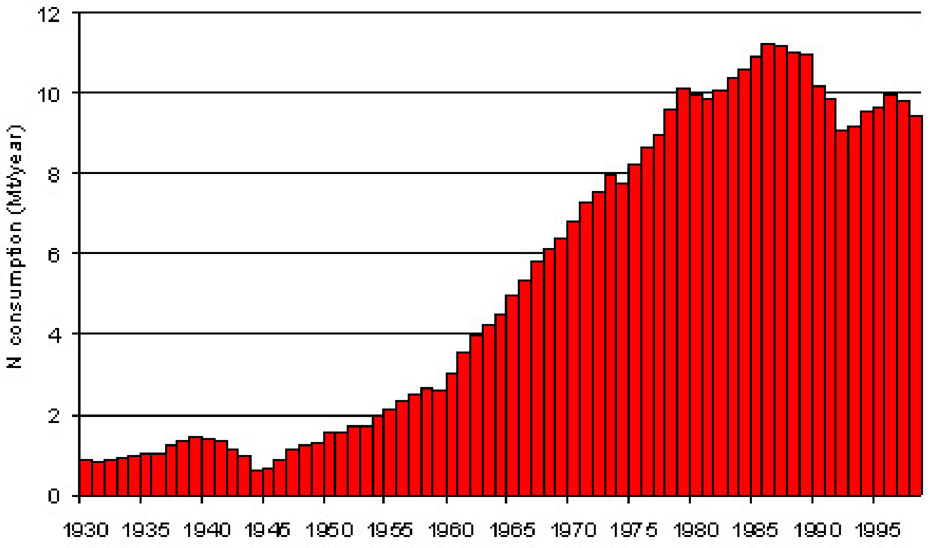

**FIGURE 2.** Development of the use of nitrogen from chemical fertilisers in the EU (Source: EFMA).

the same time, animal keeping was intensified, which resulted in the production of large quantities of organic fertilisers (manure). The development of the amounts of N produced and used in the EU member states between 1993 and 1997 is presented in Table 2. In this table, data are provided for the situation before and after the expansion of the EU with 3 member states (Austria, Finland, Sweden) at the end of the last century. These data show that no recent reduction of the nitrogen "pressure" from agriculture as EU level occurred during the last decade of the past century.

The total input of nitrogen is larger than the crop uptake in many EU regions. In Table 3, the top 25 of the European regions with the highest nitrogen surplus (mineral fertilisers + organic fertilisers + deposition – fixation – harvested crops) is presented.

The N surplus in the south and east of the Netherlands is by far the highest in Europe (> 300 kg N/ha/year). But also in the other Dutch regions, Flanders, Denmark, and some German and French regions, the N surplus amounts to over 100 kg N/ha/year. A significant reduction in the N surplus between 1993 and 1997 was observed in the Netherlands (south, east) and in Denmark, whereas an increase occurred in Finnish regions and in Brittany, France.

## ACTION PROGRAMMES

Since agriculture can be regarded as a diffuse source of nitrate pollution, mitigation options have to be mainly based on generic measures. Action programmes are mandatory for all NVZs. Often, national authorities provide a framework action programme for all NVZs in the country, leaving room for further detailing by the local or regional authorities for local NVZs. The EU has provided an overview of measures that have to be addressed in action programmes. They are summarised in Table 4. Most of the measures are "mean oriented", leaving little room for interpretation. A further detailing of these measures, including a science-based set of basic values (e.g., the width of buffer strips related to the soil conditions and slope, the minimal periods when

**TABLE 2**
**Amounts of Mineral and Organic Nitrogen Used in the EU Member States (in Mton of N Per Year) between 1993 and 1997**

| | 1993 | 1995 | 1997 | Change 97/93 |
|---|---|---|---|---|
| **Mineral N** | | | | |
| • 12 member states | 8.7 | 9.0 | 9.2 | +6% |
| • 15 member states | — | 9.5 | 9.7 | +1% |
| **Organic N** | | | | |
| • 12 member states | 6.4 | 6.3 | 6.3 | 0% |
| • 15 member states | — | 7.1–7.8 | 7.1–7.8 | 0% |
| **Total N** | | | | |
| • 12 member states | 15.1 | 15.3 | 15.5 | +3% |
| • 15 member states | — | 16.6–17.3 | 16.8–17.5 | +1% |

**TABLE 3**
**Top 25 of EU Regions with the Highest Nitrogen Surplus (kg N/ha/year) in 1997 and the Relative Change between 1993 and 1997 (Source: Eurostat)**

| | 1993 | 1997 | Change (%) |
|---|---|---|---|
| Netherlands – south | 452 | 389 | –14 |
| Netherlands – east | 316 | 294 | –7 |
| Belgium – Flanders and Brussels | 224 | 233 | +4 |
| Netherlands – north | 188 | 195 | +4 |
| Netherlands – west | 175 | 178 | +2 |
| Germany – Nordrhein-Westfalen | 120 | 119 | –1 |
| Germany – Niedersachsen | 110 | 112 | +2 |
| Denmark | 135 | 111 | –18 |
| France – Bretagne | 81 | 103 | +26 |
| Luxembourg | 100 | 99 | –2 |
| Spain – Cataluña | 100 | 91 | –9 |
| Greece – Attiki | 89 | 91 | +1 |
| Germany – Baden-Württemberg | 89 | 89 | 0 |
| Germany – Schleswig-Holstein | 86 | 88 | +2 |
| Germany – Bayern | 88 | 88 | +0 |
| Germany – Sachsen | 88 | 88 | –1 |
| Germany – Hessen | 84 | 86 | +2 |
| Germany – Saarland | 86 | 86 | 0 |
| Germany – Brandenburg | 87 | 85 | –3 |
| Germany – Thüringen | 84 | 85 | +1 |
| Greece – Peloponnisos | 80 | 82 | +2 |
| Germany – Sachsen-Anhalt | 79 | 80 | +1 |
| Finland – Ita-Suomi | 68 | 79 | +16 |
| Finland – Pohjois-Suomi | 71 | 77 | +9 |
| Germany – Rheinland-Pfalz | 74 | 76 | +2 |

**TABLE 4**
**Overview of Potential Action Programme Measures**

| No. | Measure | Examples |
|---|---|---|
| 1 | Period of prohibition of fertiliser application | From 1 October to 1 March |
| 2 | Restrictions for application on sloped soils | Not when slope >6% |
| 3 | Restrictions for application on soaked, frozen, or snow-covered soils | Not allowed in these situations |
| 4 | Restrictions for application near watercourses (including buffer strips) | 5–10 m when slope <6%<br>10–20 m when slope >6% |
| 5 | Effluent storage works | Sufficient storage capacity |
| 6 | Capacity of manure storage | 6–10 months (related to measure 1) |
| 7 | Rational fertilisation (e.g., splitting fertilisation, limitations) | Split fertilisation for corn |
| 8 | Crop rotation, permanent crop maintenance | Winter crops |
| 9 | Vegetation cover in rainy periods, winter | Catch crops (winter) |
| 10 | Fertilisation plans, spreading records | Soil analysis |
| 11 | Other measures | Good irrigation practice |
| 12 | Date for application limits of 210 and/or 170 kg N from organic manure | <1998 for 210 kg N<br><2002 for 170 kg N |

manure application is forbidden) is currently prepared and will be carried out in the coming years.

Besides compulsory measures, also voluntary-based measures like frequent sampling and analysis of manure and subeconomic fertilisation are stimulated. Furthermore, the action programmes can also include activities employed to support regional initiatives for implementation of the action programme (e.g., through intensive extension and advice to farmers) Table 5 shows an overview of measures taken in an action programme in one of the EU-15 member states. The table also shows the result of the judgement of each measure in terms of compliance with the directive.

The evaluation of this action programme shows that it is to a large extent in compliance with the Nitrates Directive. Pollution of groundwaters from high inputs to crops is prevented to a high level (rational fertilisation measures, restricted periods for fertiliser application), in conjunction with other application restrictions (including fertiliser application on snow-covered and frozen soils). Buffer strips and restrictions for fertiliser application near watercourses will prevent direct pollution of groundwaters. This action programme could be further improved by a more tightened restriction of fertiliser application on sloped soils, further enhancement of permanent crops, and increased areas of winter cover crops.

**TABLE 5**
**Example of Action Programme Measures for all Nitrate Vulnerable Zones in One of the EU Member States**

| Measure | Details in Action Programme | Evaluation |
|---|---|---|
| Period of prohibition of fertiliser application | • 15 October to 15 April<br>• Dry/melted soil: until 15 November and from 1 April providing no surface runoff to waters<br>• Grass: always prohibited after 15 September | J |
| Restrictions for application on sloped soils | • Never when slope >10% | K |
| Restrictions for application on soaked, frozen, or snow-covered soils | • Not on water-saturated, frozen, or snow-covered soils | J |
| Restrictions for application near watercourses (buffer strips) | • 1–3 m<br>• 5–10 m when >2% slope | K / J |
| Effluent storage works | • In waterproof storage | J |
| Capacity of manure storage | • 12 months minus grazing period | J |
| Rational fertilisation (e.g., splitting fertilisation, limitations) | • Maximum manure N use in autumn<br>• Maximum N-total applied on different crops<br>• Nutrient balance of soils<br>• Cereals: 30 + 170 kg N (split gift) | J |
| Crop rotation, permanent crop enhancement | • No measures | L |
| Vegetation cover in rainy periods, winter | • 30% of arable land covered in winter (south of the country) | K |
| Fertilisation plans, spreading records | • Soil fertility and manure analyses every 5 years<br>• Bookkeeping of N fertilisation and fields | J |
| Other measures | • Farm Environmental Management plan<br>• <1.5 LU/ha<br>• Incorporation of manure in autumn within 24 h | J |
| Dates for application limits:<br>• 210 kg N/ha/year | | |
| • 170 kg N/ha/year | • May 1998 (includes N from animals during grazing) | J |

*Note:* Legend: L = poor; K / L = major improvements needed; K = revision needed; K / J = rather good; J = in compliance.

## Regional Initiatives to Support Action Programmes

There appears to be a growing awareness of the relevance of measures to prevent water pollution through nitrates for sustainable agriculture. An increasing number of additional regional activities are developed and implemented to support action programmes.

### *Ferti-Mieux*

The French initiative Ferti-Mieux, French for “improved fertilisation”, was launched in 1991 as a programme of advice to farmers for a rational fertilisation. It had its basis in the Nitrates Directive that was published in the same year. Different from legal measures, Ferti-Mieux aims at the willingness of farmers to take care of the (water) environment and is therefore based on voluntary measures. By now, it covers 1.3 million ha (22,000 farmers) spread over the entire country.

Specific elements are:

- commitment to modify existing farming practices
- a collective approach on preservation of the water quality in the catchment area
- scientific support (including advisory services)
- continuous monitoring and evaluation of new practices
- active communication between farmers and the advisors

**Table 6 summarises important results of some regional Ferti-Mieux activities.** These data indicate that significant reductions of input and surplus of N can be achieved under practical conditions, with a potential of major reductions of nitrate concentrations in groundwaters.

### *Baden-Württemberg, Germany*

Around 80% of the drinking water in Baden-Württemberg originates from groundwater. The government aims at an increase of the water protection area from 21% of the land area to 27% in the next years. The first initiative dates back to 1976 (investment programme for slurry storage) and 1988 (decree to protect catchment areas). Furthermore, improved manure application was enforced by 1995. Recent initiatives aim at an increase in on-farm advice, and soil analysis to improve fertilisation plans. Other, obligatory, measures are:

- increase of the area with permanent grassland
- no N fertilisation outside the growing season
- reduction by 20% of the advised N dosage on crops
- reduced soil-tillage activities in autumn

To support the activities, 75 advisors are present to guide the farmers, and 55,000 to 80,000 soil samples are taken on various depths (0–30, 30–60, and 60–90 cm) to support the fertilisation practices. Results provided by the authorities show that the nitrate–N content in the soil of the catchment area was

**TABLE 6**
**Some Important Results of Regional “Ferti-Mieux” Activities in France**

| | Region | | |
|---|---|---|---|
| | Source de Gorze | Haute Saintois | Seine-et-Marne |
| Area (×1,000 ha): | | | |
| • Total | 5.9 | | 200 |
| • Agricultural | 4.0 | 0.8 | 120 |
| • Permanent pastures | 0.5 | | |
| Number of farmers | 56 | 30 | 1,200 |
| N input status: | *Input on regional level:* | *Surplus on field level:* | *Input on field level:* |
| • Initial input/surplus | 0.7 kton N | 113 kg/ha/year | 130 kg/ha/year |
| • Realised input/surplus | 0.5 kton N (= –30 | <100 kg/ha/year | <100 kg/ha/year |
| • Reduction goal (%) | kg/ha/year) | | |
| • Realised reduction (%) | 20 | | |
| | 23 | | |
| Catch crops | Yes | Yes | No |
| Splitting fertilisation | Yes | No | Yes |
| Soil analysis | No | No | Yes |
| Impact on water quality (nitrate concentration) | Decrease from 60 to 45 mg/l between 1992 and 1999 | Still under survey | Still under survey |

**TABLE 7**
**Results of the Thessaly Project Against Nitrates Pollution: Newly Recommended N Fertilisation Levels for the Main Crops and Reductions Achieved**

| Crop | Original Recommendations (kg N/ha/year) | Reduction of N Fertilisation (%) | Improved Recommendations (kg N/ha/year) |
|---|---|---|---|
| Cotton | 125–160 | 25–35 | 95–105 |
| Corn | 260–305 | 20–25 | 210–230 |
| Wheat | 130–160 | 10–20 | 120–130 |
| Sugarbeet | 125–165 | 25–30 | 95–115 |
| Tomato | 245–290 | 20–25 | 195–215 |

reduced by 40–60% between 1988 and 1999, depending on the crop type (e.g., wheat from 60 to 30 kg N/ha and corn from 120 to 40 kg N/ha).

### *Thessaly, Greece*

Results of the pilot nitrogen pollution project in Thessaly, operated within the framework of the National Action Programme, has indicated that farmers have started altering their attitude on crop fertilisation towards a rational and more scientific approach. So far, they have experienced that a reduced amount of nitrogen does not necessarily correspond to a yield reduction, especially for cotton. Nitrogen uptake is low during the initial growing stages and thus nitrate losses can be considerably diminished with a reduced presowing nitrogen fertilisation. Farmers are now convinced that the nitrogen fertilisation efficiency is increased and can be succeeded by splitting the recommended amount to a number of doses.

In the project against nitrate pollution in Thessaly, more than 3,200 farmers participated for the period 1996–2000. It was estimated that a reduction of the applied N fertilisers of about 10 kton for the pilot area of Thessaly was achieved. The main results are summarised in Table 7.

These data demonstrate that a close interaction between research, government, and farmers is a promising way of successful implementation of measures that lead to a significant reduction of the input of N in agriculture and consequently also in the losses of nitrogen through nitrate leaching.

### Impact of Action Programmes

The goal of the water-quality monitoring activities that member states have to conduct in the framework of the directive is to assess the impact of the directive and the action programmes. Preliminary data show that nitrates concentrations in ground and surface waters stabilised during the past monitoring period. However, the results can be biased due to insufficient harmonisation in the protocols (e.g., sampling depths) for measurements. Furthermore, the implementation of action programmes has not yet reached a satisfactory level and appears to be slow in many member states. Finally, the N input through mineral and organic fertilisers did not significantly change during the previous decade. A major improvement in these areas is needed in order to expect, through action programme implementation, and see, through monitoring, an impact of the directive.

## CONCLUDING REMARKS

The agriculture-related nitrogen pressure to the EU aquatic environment has strongly increased after the 2nd World War. As a consequence, nitrate concentrations in groundwater and surface water has drastically increased. The EU Nitrates Directive aims at a reduction of the nitrate concentration in groundwaters to values below 50 mg/l. To support this aim, Nitrate Vulnerable Zones were designated in all 15 member states. Moreover, member states were forced to implement action programmes with mandatory measures to reduce nitrogen pressures. Member states increasingly appear to comply with the Nitrates Directive, which fact is, among other ways, illustrated by the various regional activities to promote and support action programs. Increased activities with respect to monitoring protocols and action programme implementation are necessary to see results in terms of reduced nitrate concentrations in ground waters.

| | | |
|---|---|---|
| Received: | July | 23, 2001 |
| Revised: | November | 9, 2001 |
| Accepted: | November | 9, 2001 |
| Published: | December | 12, 2001 |

## BIOSKETCH

**Tommy Dalgaard** is a Scientist with the Danish Institute of Agricultural Sciences in Denmark. In 1996 he received his M.Sc. in agroecology, and in 2001 completed his Ph.D. thesis, Simulation and Generalisation of Agricultural Resource Use. His work focuses on agricultural systems analyses. The aim is to integrate data in Geographical Information Systems, and to use these detailed data for bottom-up simulation and mapping of indictors for agricultural production. Also interactions with environment, economy, nature and society are included in the analysis. He has been an invited speaker at the International workshop, "Dissipation of N from the Human N-Cycle, and its Role in Present and Future $N_2O$ Emissions", Oslo (co-sponsored by the Intergovernmental Panel on Climate Change, IPCC), and at the EU-statistics seminar, "How Can Agricultural Statistics Meet Environmental Information Needs", Copenhagen. Dr. Dalgaard has been an invited expert for The Danish Parliament's committee for Nature and Environment, "Hearing on Loss of Nutrients to the Aquatic Environment", and "2nd European Invitational Expert Seminar on Life Cycle Assessments of Food Products", The Hague, The Netherlands.
